# Atroxlysin-III, A Metalloproteinase from the Venom of the Peruvian Pit Viper Snake *Bothrops atrox* (Jergón) Induces Glycoprotein VI Shedding and Impairs Platelet Function

**DOI:** 10.3390/molecules24193489

**Published:** 2019-09-26

**Authors:** Luciana S. Oliveira, Maria Inácia Estevão-Costa, Valéria G. Alvarenga, Dan E. Vivas-Ruiz, Armando Yarleque, Augusto Martins Lima, Ana Cavaco, Johannes A. Eble, Eladio F. Sanchez

**Affiliations:** 1 Research and Development Center, Ezequiel Dias Foundation, 30510-010 Belo Horizonte, MG, Brazil; luciana.oliveira@funed.mg.gov.br (L.S.O.); valeria.alvarenga@funed.mg.gov.br (V.G.A.); 2 Institute of Physiological Chemistry and Pathobiochemistry, University of Münster, 48149 Münster, Germany; mina.estevao@gmail.com (M.I.E.-C.); acmcavaco@gmail.com (A.C.); johannes.eble@uni-muenster.de (J.A.E.); 3 Laboratorio de Biología Molecular-Facultad de Ciencias Biológicas, Universidad Nacional Mayor de San Marcos, Av. Venezuela Cdra 34 S/N, Ciudad Universitaria, Lima 01, Lima 14-0576, Peru; dvivasr@unmsm.edu.pe (D.E.V.-R.); ayarlequec@unmsm.edu.pe (A.Y.); 4 Laboratory of Hemodynamics and Cardiovascular Technology, École Polytechnique Fédérale de Lausanne, 1015 Lausanne, Switzerland; augusto.martinslima@epfl.ch

**Keywords:** snake venoms, metalloproteinase/disintegrin, extracellular matrix, atroxlysin-III, platelets, glycoprotein VI

## Abstract

Atroxlysin-III (Atr-III) was purified from the venom of *Bothrops atrox*. This 56-kDa protein bears N-linked glycoconjugates and is a P-III hemorrhagic metalloproteinase. Its cDNA-deduced amino acid sequence reveals a multidomain structure including a proprotein, a metalloproteinase, a disintegrin-like and a cysteine-rich domain. Its identity with bothropasin and jararhagin from *Bothrops jararaca* is 97% and 95%, respectively. Its enzymatic activity is metal ion-dependent. The divalent cations, Mg^2+^ and Ca^2+^, enhance its activity, whereas excess Zn^2+^ inhibits it. Chemical modification of the Zn^2+^-complexing histidine residues within the active site by using diethylpyrocarbonate (DEPC) inactivates it. Atr-III degrades plasma fibronectin, type I-collagen, and mainly the α-chains of fibrinogen and fibrin. The von Willebrand factor (vWF) A1-domain, which harbors the binding site for GPIb, is not hydrolyzed. Platelets interact with collagen via receptors for collagen, glycoprotein VI (GPVI), and α2β1 integrin. Neither the α2β1 integrin nor its collagen-binding A-domain is fragmented by Atr-III. In contrast, Atr-III cleaves glycoprotein VI (GPVI) into a soluble ~55-kDa fragment (sGPVI). Thereby, it inhibits aggregation of platelets which had been stimulated by convulxin, a GPVI agonist. Selectively, Atr-III targets GPVI antagonistically and thus contributes to the antithrombotic effect of envenomation by *Bothrops atrox*.

## 1. Introduction

Bioactive snake venom compounds have evolved from convergent or divergent evolution [1,2,3]. Due to their high efficacy, several venom proteins/peptides, which interfere with hemostasis by affecting coagulation and/or platelet function, have been harnessed in the diagnosis and treatment of hemostatic disorders [4,5,6,7]. Snake venom metalloproteinases (SVMPs) account for more than 30% of venom proteins in most Viperidae venoms and hence majorly contribute to the symptoms of viperid envenomation [8,9]. SVMPs are classified according to their domain organization into P-I to P-III classes [10,11,12]. Class P-I (P-I) is composed of a metalloproteinase (M) domain of about 200 amino acid residues [13,14]. P-II and P-III SVMPs have an additional disintegrin (-like) (D) domain and cysteine-rich (C) domain, in addition to the M domain. P-III group are further classified into sub-classes based on their different post-translational modifications, such as proteolytic processing between the M and D domains (P-IIIb) or dimerization (P-IIIc). The multimeric P-IV class of SVMPs (formerly P-IV), containing an additional snake C-type lectin-like proteins (snaclecs) [10,11,12,13,14,15], has meanwhile been included in the P-III class as a subclass (P-IIId) since no mRNA transcript has been found until now. The criterion for this classification was based on the presence or absence of several non-catalytic domains as evidenced via mRNA transcripts and proteins purified in the venom [11]. The P-III SVMPs exert strong hemorrhagic activities, likely because the M-domain is supported by the non-catalytic D and C domains. They harbor several substrate binding sites (exosites) in the disintegrin loop and hyper-variable region (HVR) [15,16]. Moreover, disintegrins and P-III SVMPs antagonize platelet aggregation by preventing αIIbβIII integrin from the binding of fibrinogen and the von Willebrand factor (vWF) [17,18].

Sharing the homologous structure of their catalytic domain, SVMPs, together with members of the ADAMs (a disintegrin and metalloproteinase) protein subfamily and ADAMTS (a disintegrin and metalloproteinase with thrombospondin motif) as well as with matrix metalloproteinases (MMPs) form the M12 clan of metalloproteinases (Merop database: http://merops.sanger.ac.uk/) [10,11,12,16]. These proteinases degrade several essential proteins, such as plasma proteins, extracellular matrix (ECM) components, and blood coagulation factors. They also affect platelet inter alia by cleaving their adhesion receptors [14,15,16,17,18], among which glycoprotein (GP) GPVI and GPIbα of the GPIb-IX-V complex are crucial for thrombus formation at arterial shear rates [19,20,21]. GPVI is a collagen-binding platelet receptor that mediates platelet adhesion and aggregation at relatively low physiological shear stress while the vWF-receptor, GPIb-IX-V, elicits platelet aggregation under high shear conditions in arterioles and stenotic arteries [19,21]. Acurhagin from *Deinagkistrodon* (formerly *Agkistrodon*) *acutus* venom antithrombotically targets GPVI and collagen [22]. Jararhagin, from *B. jararaca* venom, inhibits collagen-induced platelet aggregation by non-enzymatically interfering with the collagen-α2β1 integrin interaction and by proteolytically cleaving the β1 subunit on the platelet membrane [18,23]. Other studies indicated that the P-III SVMPs alborhagin (*Trimeresurus albolabris*) and crotarhagin (*Crotalus horridus horridus*) induced GPVI proteolysis by a mechanism that involves platelet stimulation, and activation of endogenous platelet sheddases [24].

*Bothrops* (lanceheads) are neotropical pit viper snakes of medical relevance, which are widely spread throughout the tropical and non-tropical regions of Central and South American countries [8,25]. *B. atrox* is responsible for approximately 83% of all recorded snake bites throughout the Amazon region and thus surpasses any other South American venomous snake [25,26,27]. Furthermore, the vast majority of notified snake bites (~85%) in the tropical rainforests regions of eastern Peru are due to *B. atrox* [13,28]. The most clinical manifestations of *B. atrox* bite are local tissue damage and/or severe systemic hemorrhages, and may vary in different regions of the Amazonian range [25,26,27]. These detrimental effects are induced mostly by SVMPs such as the P-I class toxin termed atroxlysin-I (Atr-I) previously characterized in our laboratory [13]. Here we report a novel P-III SVMP from *B. atrox* venom. This is, to our knowledge, the first report on the isolation and biochemical characterization of a high molecular mass hemorrhagic metalloproteinase from Peruvian *B*. *atrox* venom. We propose the name atroxlysin-III (Atr-III) considering, the original source (atrox-) and the proteolytic action (-lysin) of the P-III class metalloproteinase. Its complete amino acid sequence was deduced from the corresponding cDNA. Our results of its biochemical and functional properties reveal the molecular mechanism of Atr-III on platelet function, hemostasis, and thrombosis.

## 2. Results

### 2.1. Purification of Atroxlysin-III

**Figure 1:**
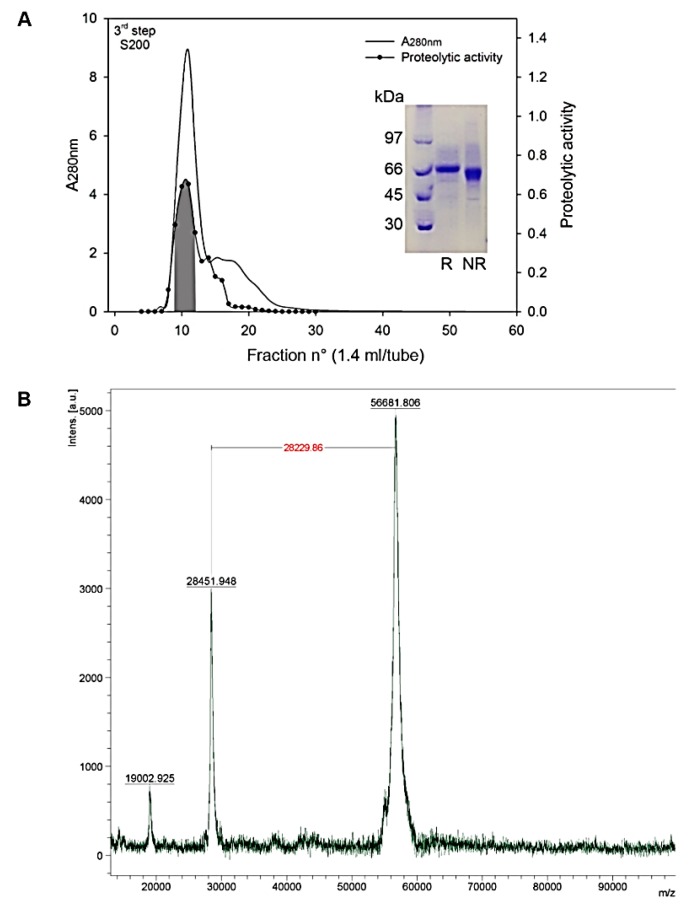
Purification of Atroxlysin-III from *B. atrox* venom (3rd step). (**A**) Fraction from the second step (DEAE-ion exchange column) containing hemorrhagic activity was applied to a Sephacryl 200 column. The column was equilibrated and eluted with 50 mM Hepes buffer, pH 8.0, containing 0.1 M NaCl, 2 mM CaCl_2_ at a flow rate of 3.4 mL/h. Active fractions containing Atr-III were pooled (area under curve highlighted in grey). The inset indicates the SDS-PAGE (12% gel) of purified Atr-III under reduced (R) and non-reducing (NR) conditions. (**B)** Mass spectrometry of native Atr-III. Purified Atr-III was analyzed by MALDI TOF/TOF. The main signal (displayed in arbitrary units—a.u.) corresponded to a singly charged ion of 56,681.8 daltons.

The progress of purification was followed by determining the proteolytic activity on dimethylcasein (DMC), fibrinolytic activity, coagulant activity on fibrinogen (Fg) and by measuring the hemorrhagic effect of each fraction in vivo. First, gel filtration on a Sephacryl S-200 column separated the crude venom into eight peaks (P1 to P8, Figure S1). Proteolytic and hemorrhagic activity were concentrated in peaks P1 and P4, representing high and low Mr proteins of <50 and ~23 kDa. The latter contained the previously described Atr-I, a P-I class SVMP [13]. Peak P1 contained the hemorrhagic metalloproteinase of P-III class and was further separated by a DEAE Sepharose column into five peaks (A, B, C, D, and E; Figure S1). Peak C contains the hemorrhagic and proteolytic activity and showed a major band in SDS PAGE with only minor contaminants. Further purification with a Sephacryl S-200 column (Figure 1A) yielded an active proteinase with mol. mass of approximately 56 and 60 kDa under reducing and non-reducing conditions, respectively (Figure 1, inset). MALDI-TOF-TOF revealed its mass to be 56,681.8 Da (Figure 1B). The characteristic proteolytic and hemorrhagic properties of Atr-III are presented in Table 1.

### 2.2. Nucleotide Sequencing of Atroxlysin-III cDNA

**Figure 2:**
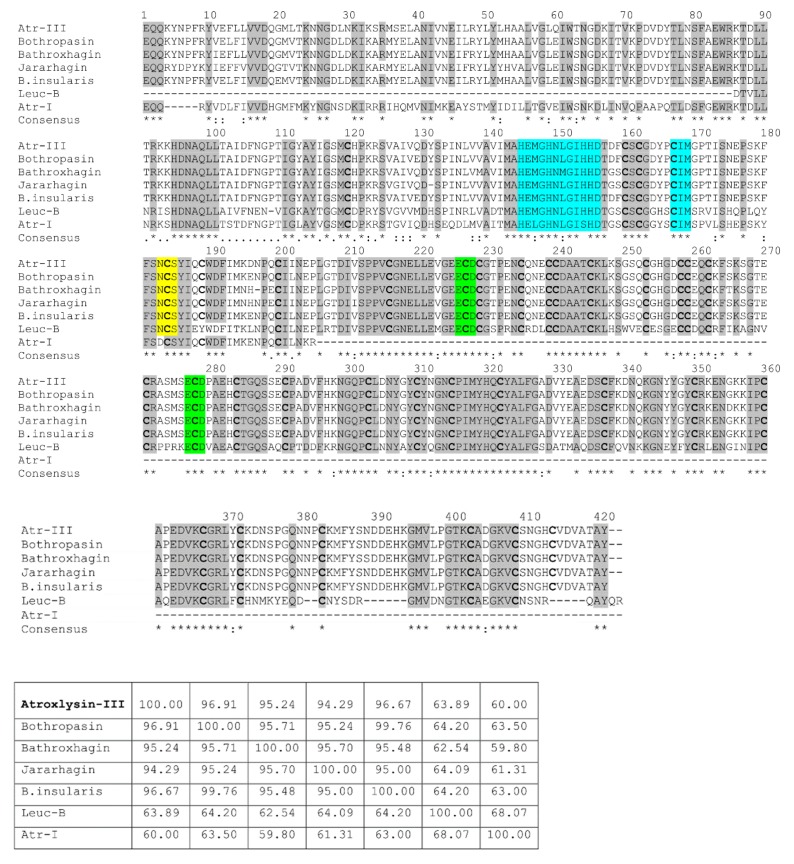
Sequence alignment of Atr-III with other snake venom metalloproteinases (SVMPs) from different pit viper species. The sequence of Atr-III is aligned corresponding to those of the following venom proteins: Bothropasin from *B. jararaca* (AAC61986), Batroxrhagin from the Brazilian *B. atrox* (KR632593), Jararhagin from *B. jararaca* (P30431), *B. insularis* P-III from *B. insularis* (AAM09693), Leuc-B from *B. leucurus* (P86092) and Atr-I, a P-I SVMP from the same *B. atrox* venom (P85420). Amino acid numbering follows that of Atr-III. Residues are denoted if highly homologous (*) or strongly similar (:). The zinc binding motif (HEGNHLGIHHD) and the methionine 168 of the Met-turn region are invariant and are highlighted in blue. Putative N-linked glycosylation site is bold and boxed in yellow. The cysteine residues are bold and highlighted. Disintegrin-like (ECD) sequences are highlighted in green. Gaps (-) were introduced to optimize the sequence identity. a.a., amino acids. Sequences were aligned using the CLUSTAL W Program.

The primary structure of Atr-III was deduced from the corresponding cDNA isolated from the total RNA of *B. atrox* snake venom. Using RT-PCR, 30 ng/μL cDNA were synthesized from 150 μL of freshly milked venom from one specimen. The fully assembled cDNA sequence and deduced amino acid sequence (Figure S2) revealed that pre-pro-atroxlysin-III consists of a signal sequence, a pro-domain, a metalloproteinase domain, a disintegrin-like domain and cysteine-rich domain. The signal peptide, MIHVLLVTICLAAFPYQG, is cotranslationally cleaved from the Atr-III precursor. The pro-domain harbors the activity-regulating cysteine-switch motif, PKMCGVT. After the activating cleavage of the pro-domain, a pyroglutamate residue, similar to other P-III SVMPs, forms the N-terminus of the mature protein, which comprises 420 amino acids and consists of a metalloproteinase (M), a disintegrin-like (D) and a cysteine-rich (C)-domain. The primary structure of Atr-III shares high sequence homology with other SVMPs family members including jararhagin [29], bothropasin [30], batroxrhagin [31], MP-III *B. insularis* [32], Leuc-B [33] and the P-I class Atr-I [13] from the same *B. atrox* venom (Figure 2). The sequence identities of Atr-III precursor with bothropasin, MP-III *B. insularis*, jararhagin, batroxrhagin, and Leuc-B are 96.9%, 95.2%, 94.3%, 96.7%, and 63.9%, respectively (Figure 2). The M domain contains the typical zinc-chelating motif, HEMGHNLGIHHD from residues 144 to 155 as well as the methionine-turn motif, CIM, that is also involved in zinc-binding of the metzincin superfamily of metalloproteinases [10,11] (Figure 2). The D-domain contains a putative integrin-interacting motif ECD in the corresponding D-domain sequence that is homologous to DCD, a putative collagen binding site, which is characteristic for many P-III SVMPs. A potential Asn-linked sugar chain glycosylation motif was found at Asn183 in the M-domain. Similarly, it is also found in the corresponding regions of the other five P-III SVMPs (bothropasin, batroxrhagin, jararhagin, MP-III *B. insularis* and Leuc-B).

### 2.3. Three-dimensional Model and Phylogenetic Study

**Figure 3:**
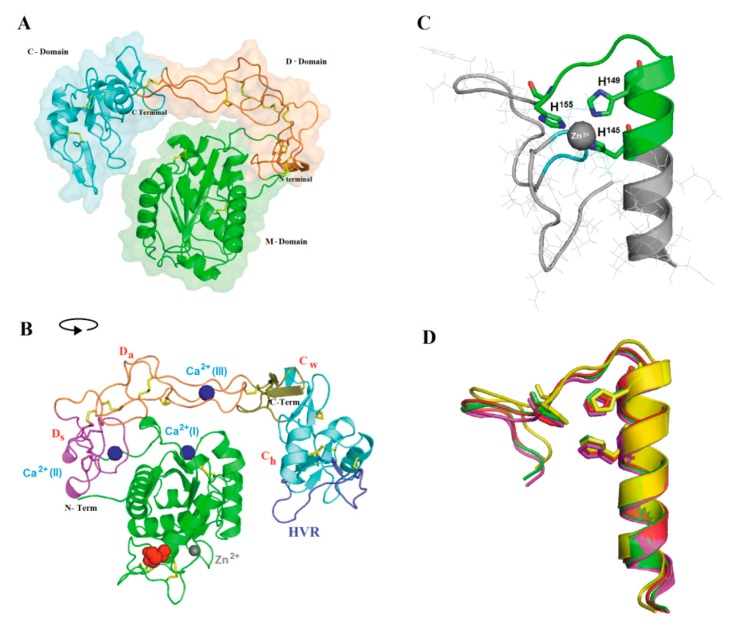
Theoretical Atroxlysin-III 3D model. (**A**) A homology model of Atr-III generated with Modeller 9.13 using crystal structures of other P-III SVMPs as templates. The metalloproteinase (M), disintegrin-like (D) and Cysteine-rich (C) domains are indicated by green, orange, and cyan colors, respectively. Elements of secondary structure α-helices, β-structures, N- and C-terminal ends are presented. Disulfide bonds are in yellow sticks. (**B**) Principal motifs of Atr-III according to Igarashi et al. [34]. The M-domain, Ds, Da, Cw, and Ch segments and the hyper-variable-region (HVR) are shown in green, magenta, orange, olive, cyan, and blue, respectively. Zinc and calcium ions are represented as grey and blue spheres, respectively. N-glycosylation motif is indicated in red spheres. (**C**) Details of the zinc binding site of Atr-III where the positioning of the three histidine residues are shown. (**D**) Superposition of the zinc-binding residues of Atr-III with the P-III SVMPs are shown in sticks and labeled. *B. jararaca* (red), *C. atrox* (magenta), and *A. acutus* (yellow). For interpretation of the references to color in this figure legend, the reader is referred to the web version of this article.

**Figure 4:**
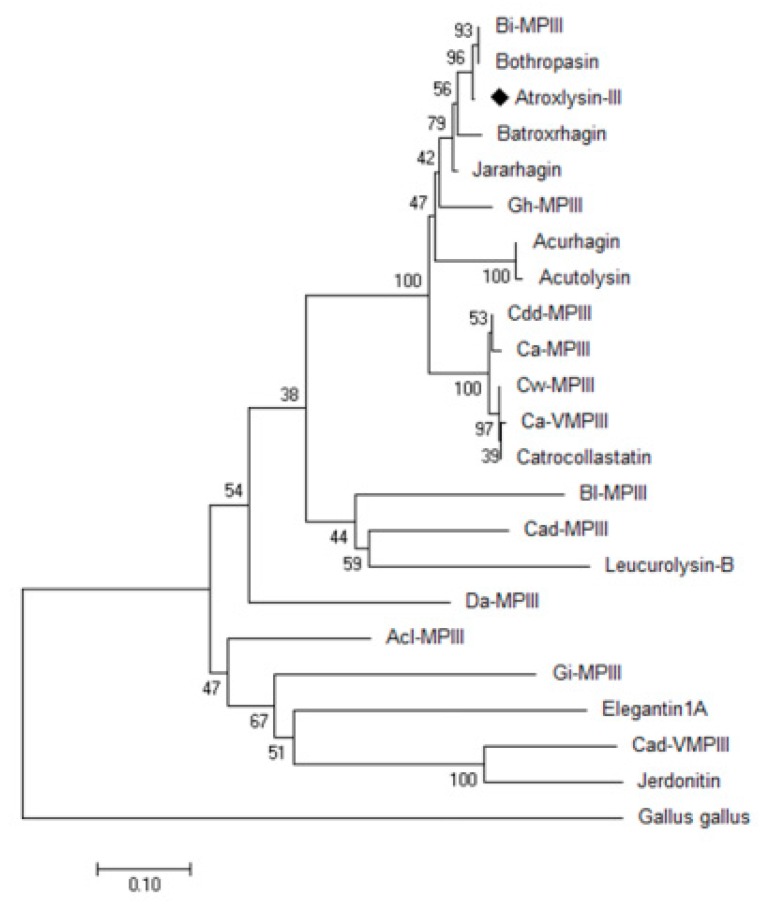
Phylogenetic tree of the P-III SVMPs. The amino acid sequences of 22 SVMPs were used for phylogenetic tree construction by the program Mega 5.1. Atr-III from Peruvian *Bothrops atrox* is marked (♦) and the others P-III class SVMPs: Bi (*Bothrops insularis*, AF490534); Bothropasin (*Bothrops jararaca*, AAC61986); Batroxrhagin (Brazilian *B. atrox*, ALB00542); Jararhagin (*B. jararaca*, CAA48323); Gh-MPIII (*Gloydius halys*, AAD02652); Acurhagin (*Deinagkistrodon acutus*, AAS57937); Acutolysin (*Deinagkistrodon acutus*, AAD27891); Cd MPIII (*Crotalus durissus durissus*, ABA42117); Ca-MPIII (*Crotalus atrox*, A4PBQ9); Cvv-MPIII (*Crotalus viridis viridis*, ACV83933); Ca-VMPIII (*Crotalus atrox*, ACV83931); Catrocollastatin (*Crotalus atrox*, AAC59672); Bl-MPIII (*Bothriechis lateralis*, AGY49227); Cad MPIII (*Crotalus adamanteus*, J3S830); leucurolysin-B (*Bothrops leucurus*, P86092); Da MPIII (*Deinagkistrodon acutus*, BAO23490); Acl-MPIII (*Agkistrodon contortrix laticinctus*, ACV83929); Gi-MPIII (*Gloydius intermedius*, AJI77146); elegantin1A (*Protobothrops elegans*, BAB69657); Ca-VMPIII (*Crotalus adamanteus*, AEJ31986); Jerdonitin (*Protobothrops jerdonii*, AAQ63966); and *Gallus gallus* ADAM9 was used as the outgroup (NP-001026567).

A three-dimensional model of Atr-III (Figure 3) was generated by the homology modeling software Modeller using crystal structures of *B. jararaca*, *C. atrox* and *A. acutus* P-III class SVMPs as templates (PDB codes: 3DSL-A, 2DW0-A and 3HDB-A, respectively). A comparison of the generated model with VAP2B [34] suggested that the disintegrin-like (D-) domain is structured into subdomains, Ds and Da. Analysis of the metal binding sites indicated that the structural model present sites for Ca^2+^ and Zn^2+^ binding and are in homology with the used templates. The Ds-domain protrudes from the M-domain close to the Ca^2+^-binding site I but distantly from the catalytic crevice. The comparison of 22 homologous P-III class SVMPs rendered a phylogenetic tree, in which the viperid metalloproteinases are separated in three main branches (Figure 4).

### 2.4. Biochemical Features of Atr-III

**Figure 5:**
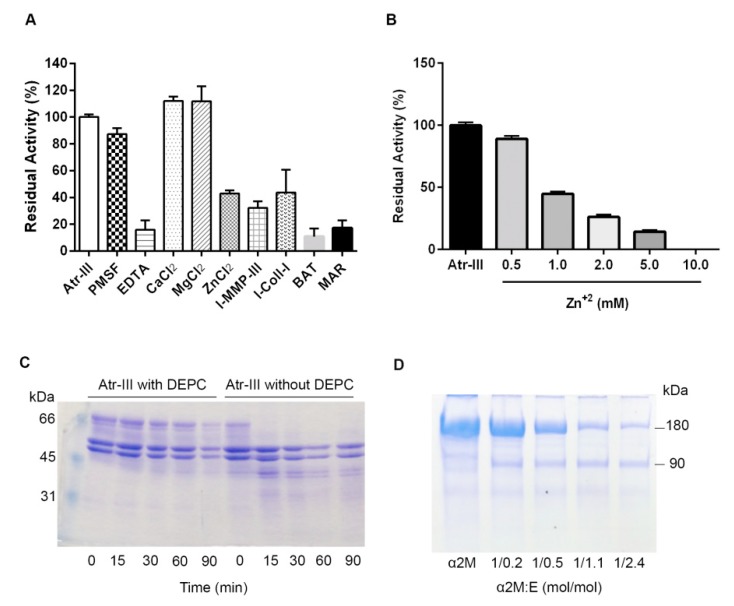
Biochemical properties of Atr-III. (**A**) Effect of several reagents on the proteolytic activity of Atr-III. The isolated Atr-III (1 μg) was treated with each reagent as described in the experimental procedures. The remaining activity was measured with DMC as substrate. (**B**) Atr-III (1 μg) was preincubated with Zn^2+^ at the indicated concentrations for 20 min, pH 7.3 at 37 °C. The remaining activity was tested with DMC. (**C**) Fibrinogen digestion by Atr-III with and without DEPC modification as described in the experimental procedures. (**D**) Atr-III (2 μg) was incubated with α2M as described in the experimental procedures. The products of enzyme:α2M interaction were analyzed by SDS-PAGE. In (**A**,**B**), values represent the means ± SD (*n* = 3). (**C**,**D**) show representative results out of three similar experiments.

Digestion of DMC was employed to quantify the proteolytic activity of Atr-III Ca^2+^ (115%) and Mg^2+^ (114%) slightly increased (Figure 5A), whereas addition of Zn^2+^ decreased the activity of Atr-III in a concentration-depended way (Figure 5B). We chemically modified Atr-III with diethylpyrocarbonate (DEPC) which specifically reacts with the imidazole ring of histidines. DEPC-modified Atr-III failed to digest fibrinogen (Fg) (Figure 5C) whereas untreated enzyme completely digested the α-chain of Fg within 15 min. Hence, Atr-III is a metalloproteinase and essentially requires complexation of a zinc-ion by histidyl residues in the active site. This explains the inhibition of Atr-III activity with a chelating agent EDTA and with the synthetic metalloproteinase inhibitors, batimastat (BAT) and marimastat (MAR). Conversely, PMSF, an active-site serine proteinase inhibitor, had no effect. Proteolysis of DMC was also blocked by the specific inhibitors MMP-inhibitor-III (I-MMP-III) and collagenase-inhibitor-I (I-Coll-I) by approximately 70% and 65%, respectively (Figure 5A). In increasing concentrations, α-2 macroglobulin (α2M) (5 min at 37 °C) was cleaved by Atr-III into the characteristic 90 kDa fragment, likely due to the cleavage of the Arg696-Leu697 bond (Figure 5D). In contrast to the complete inhibition of Atr-I and other P-I SVMPs with α2M at an equimolar ratio [13,14], the proteolytic activity of Atr-III on DMC was not significantly inhibited by α2M (not shown). These in vitro data on proteolytic activity of Atr-III were in line with the hemorrhagic effect of the enzyme in mouse model.

**Figure 6:**
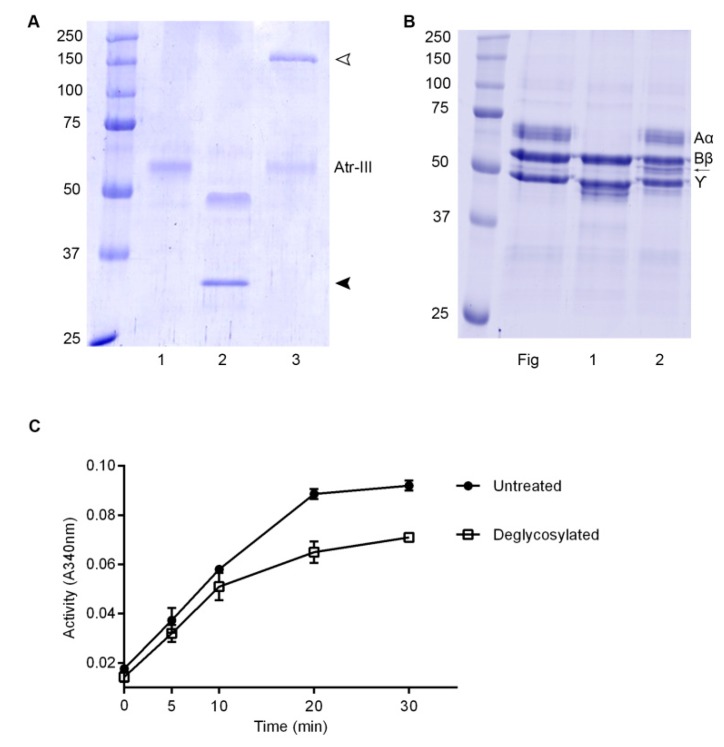
Deglycosylation of Atr-III. (**A**) SDS-PAGE (12% gel) of Atr-III untreated (1), or after treatment with PNGase F (2) or O-glycosidase (3), a band at ~35 kDa in lane 2 is an excess of PNGase F (indicated by a black arrow head) and a band at ~150 kDa is O-glycosidase (open arrow head). (**B**) Undigested fibrinogen (Fg), and Fg digested with Atr-III (1) or Fg treated with N-deglycosylated Atr-III (2). Molecular mass markers are shown in the left lanes of (**A**,**B**). (**C**) DMC hydrolysis by Atr-III (2 µg) without (-●-) or with prior treatment with PNGase F (-□-). Results are presented as SD (*n* = 3). Note that loss of N-deglycosylation affected the venom enzyme activity.

Atr-III bore N-linked glycoconjugates, a frequent property of P-III SVMPs. The presence of carbohydrate moieties on Atr-III was proven by incubating the enzyme with recombinant PNGase F. After treatment with PNGase F, Atr-III shows a 7 kDa lower Mr in SDS-PAGE under reducing conditions as compared to untreated Atr-III (Figure 6A, lane 2). In contrast, incubation with O-glycosidase did not alter the size of the proteinase (Figure 6A, lane 3). The carbohydrate moiety of Atr-III affected its stability and functional activity, as Atr-III pretreated with PNGase F under non-denaturing conditions changed its proteolytic activity on Fg and DMC. While untreated Atr-III digested the α-chain of Fg completely (Figure 6B, lane 1), digestion of Fg with deglycosylated enzyme showed an additional, yet minor protein band below the β-chain of Fg, suggesting that deglycosylation of Atr-III partially reduced its structural stability. In addition, DMC proteolysis with deglycosylated Atr-III under non-denaturing conditions revealed a partial loss of enzymatic activity in comparison with untreated enzyme (Figure 6C).

### 2.5. Effect of Atr-III on Plasma and ECM Components

**Figure 7:**
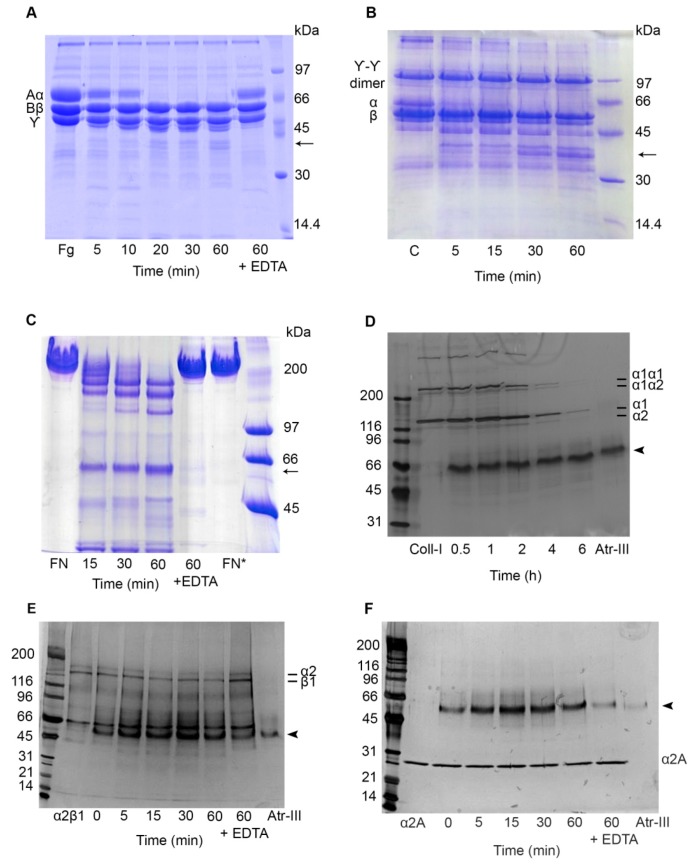
SDS-PAGE analysis of fibrinogen, fibrin, fibronectin, type I collagen, α2β1 integrin and its recombinant α2A-domain (rα2A) after digestion with Atr-III. Digestion reactions of Fg (**A**), fibrin (**B**), FN (**C**), Coll-I (**D**), α2β1 integrin (**E**) and its rα2A domain (**F**) were carried out at molar ratio of 1:150 (enzyme/substrate) for Fg, fibrin and FN, and 1:50 for collagen I for the indicated intervals at 37 °C. α2β1 integrin or its rα2A domain were incubated with 10 µg/mL of Atr-III for the same intervals at 37 °C. Reactions were stopped by addition of 10 mM EDTA. Aliquots of the incubation mixtures were analyzed by SDS-PAGE under reducing conditions in 12% gels for Fg and fibrin, 7% for FN and 5% for collagen I and 7–15% gradient gel for α2β1 integrin and rα2A domain. The positions of the three polypeptide chains of Fg control Aα, Bβ and γ (**A**) as well as fibrin control γ-γ dimer, α and β (**B**) are indicated at the left. Typical type I collagen chains (cross-linked α1α1, α1α2, chain dimers, and monomeric α1 and α2) are indicated at the right (**D**). In panels E and F, α2β1 or rα2A-domain were incubated with 10 µg of Atr-III for the indicated intervals. Reactions were stopped by addition of 10 mM EDTA and analyzed by SDS-PAGE (7–15% gradient gel). Controls of Atr-III in (**D**), (**E**) and (**F**) are indicated by black arrow heads. The results shown are representative of three similar experiments.

SVMPs generally have fibrino(geno)lytic activities and might degrade ECM proteins resulting in hemostatic dysfunction and hemorrhagic attack of the microvasculature integrity, respectively. Hence, proteolytic activity of Atr-III on Fg, fibrin, fibronectin (FN), type I collagen, IV and laminin-111 (LM), was analyzed. As the α-chains of Fg or fibrin were degraded within 5 min, Atr-III was classified as an α-fibrinogenase (Figure 7A,B). FN is degraded by Atr-III at a molar enzyme:substrate ratio of 1:100 to one major band of ~58-kDa, which was also released by the hemorrhagic P-I class Atr-I [13]. Other two degradation fragments of approximately 190 and 170 kDa, respectively, were observed within 15 min on reduced SDS-PAGE (Figure 7C). Furthermore, Atr-III hydrolyzed the fibril-forming type I collagen, as its α1α1, α1α2, and partially the α1 and α2 chains were totally digested after 4 h and 6 h (Figure 7D). In contrast, Atr-III showed only minor or undetectable in vitro degradation of laminin-111 and type IV collagen, both major components of the subendothelial basement membrane (not shown). EDTA-treatment of Atr-III completely abolished its proteolytic activity on ECM proteins, Fg (Figure 7A) and fibrin.

### 2.6. Atr-III also Targets Platelet Receptors

**Figure 8:**
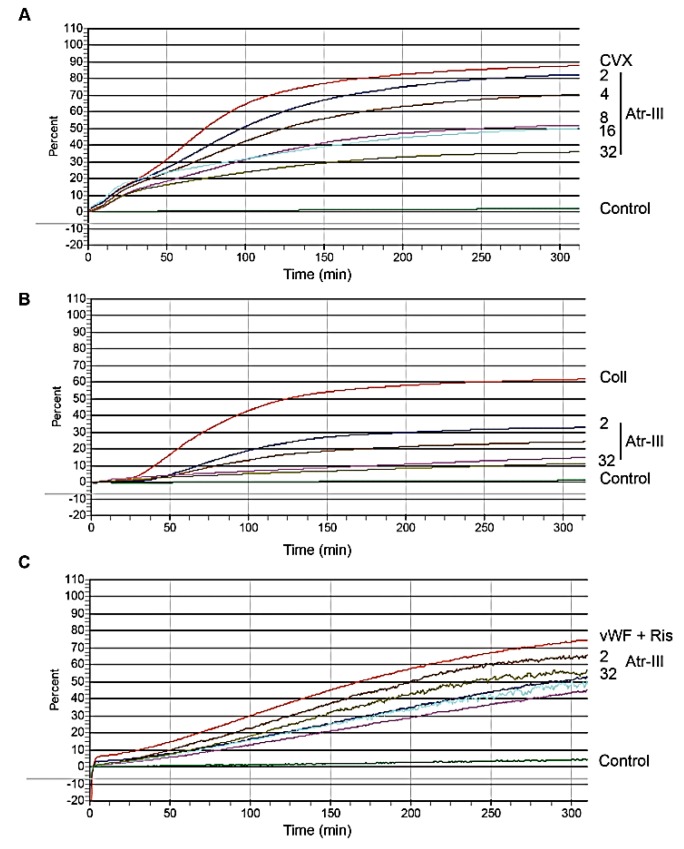
Atr-III inhibits aggregation of platelet stimulated with convulxin (CVX) (**A**) or collagen (**B**) in a concentration-dependent manner. However, it shows little effect on vWF + Ristocetin (Ris) induced aggregation (**C**). Washed human platelets (225 µL, 2.5 × 10^5^/µL) were pre-incubated with different concentrations of Atr-III (2 to 32 µg/mL) for 3 min and stirred (600 rpm) at 37 °C, and then different platelets agonists were added (CVX, 6 µg/mL), collagen-I (10 μg/mL) or vWF (5 µg/mL) + Ris (0.5 mg/mL), and platelet aggregation was recorded aggregometrically. The control response is shown by the upper tracing for each agonist. One of four similar experiments for each agonist is presented with platelets from different donors.

Platelets have a critical role in hemostasis and an emerging role in physiological processes including inflammation [19,20,21]. So far, numerous SVMPs are known for their inhibitory effects on platelet aggregation induced by several agonists. The platelet surface receptor α2β1 integrin, which triggers collagen-induced platelet aggregation was slightly cleaved within its α2 subunit after 15 min (Figure 7E). EDTA-treated Atr-III completely abolished its proteolytic activity. As the collagen-binding domain of this integrin, rα2A-domain, which appears as a monomer of ~30-kDa in SDS-PAGE was not cleaved by Atr-III, we concluded that Atr-III cleaved integrin α2β1 outside its ligand binding site (Figure 7F). Furthermore, the effect of Atr-III on GPVI, another collagen receptor of platelets, on platelet aggregation was examined by stimulation with GPVI agonists, such as convulxin (CVX) and collagen was tested by aggregometry. If platelets were pretreated for 3 min with Atr-III, the enzyme dose-dependently inhibited aggregation induced by CVX (Figure 8A and Figure S3) or collagen (Figure 8B and Figure S3) with half-maximal inhibition concentration (IC_50_) values of 0.33 and 0.20 µM, respectively. Conversely, Atr-III blocked vWF-ristocetin (Ris)-induced platelet aggregation very weakly (Figure 8C and Figure S3). Moreover, platelet aggregation induced by thrombin, which activates platelets in a different way, was only slightly delayed but not inhibited (not shown). These results suggest that signal transduction within platelets via other receptors is not affected by Atr-III.

**Figure 9:**
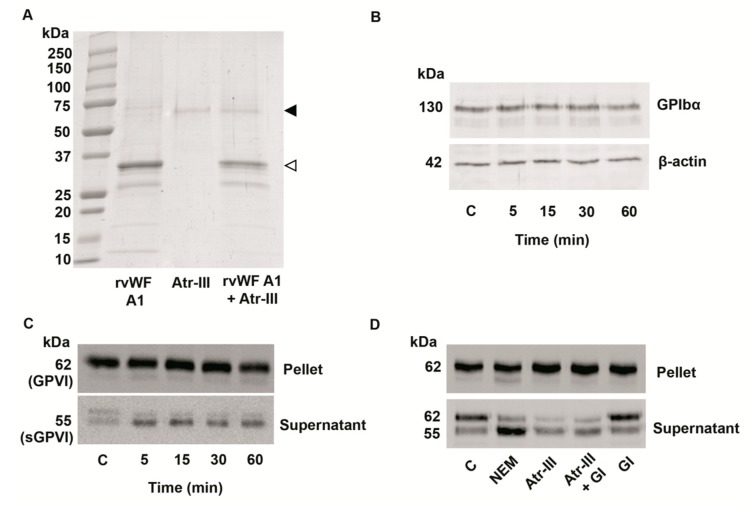
Atr-III does not cleave either the recombinant A1 domain of vWF (rvWF-A1) (**A**) nor GPIbα (**B**). (**A**) 5 µg Atr-III was incubated with rvWF-A1 (5 µg) as described in the experimental methods at 37 °C. The proteolysis of rvWF-A1 (indicated by an open arrowhead) was analyzed by SDS-PAGE (5–15%). The black arrow head indicates Atr-III. (**B**) Washed platelets were incubated with Atr-III (10 µg) at 37 °C at several intervals and the reaction was stopped by addition of SDS loading sample buffer. The platelet lysate was blotted with anti-CD42/GPIb or β-actin as endogenous control. (**C**) Platelet pellets or supernatants of washed platelets treated with Atr-III (10 µg) at 37 °C at several intervals or (**D**) NEM (10 mM), Atr-III (10 µg), Atr-III (10 µg) plus GI254023X (100 nM) and GI254023X (100 nM) alone were blotted with anti-GPVI antibodies. The position of GPVI (62 kDa) and the soluble ~55 kDa GPVI fragment are shown. C = platelet control.

In order to investigate if the venom enzyme cleaves rvWF A1-domain which appears as a band of ~35-kDa (Figure 9A) and contains the binding site for GPIb, rvWF A1-domain was incubated with Atr-III and assayed for proteolysis by SDS-PAGE. As showed (Figure 9A) the rvWF A1-domain was resistant to proteolysis by Atr-III. Furthermore, to analyze the proteolytic susceptibility of the vWF receptor, the GPIb-IX-V complex was challenged by Atr-III treatment of platelets at 37 °C, subjected to SDS-PAGE under reducing conditions and analyzed by immunoblotting with an anti-CD42/GPIbα antibody. Under our experimental conditions, GPIbα (~130-kDa, named glycocalicin) was not affected by Atr-III (Figure 9B). In addition, to investigate the mechanisms for metalloproteinase-mediated ectodomain shedding of the platelet receptor GPVI on human platelets, we used anti-GPVI antibody. Atr-III induced shedding of GPVI from the platelet surface (Figure 9C). On an immunoblot of pelleted platelets developed with an anti-GPVI antibody, a band of approximately 62-kDa indicated the appearance of intact GPVI on the platelet surface (Figure 9C, upper panel), whereas the shedded ectodomain of GPVI at ~55-kDa, termed soluble GPVI (sGPVI), was detected in the supernatant of platelets treated with Atr-III for 5 min (Figure 9C, lower panel). This sGPVI fragment was also detected in response to NEM (N-ethylmaleimide). NEM, which irreversibly reacts with a cysteinyl group within the prodomain of ADAMs, amongst them ADAM10/ADAM17, and thus activates these membrane-anchored sheddases on platelets activation. (Figure 9D). To further specify the role of ADAM10, we pretreated platelets with the specific inhibitor to ADAM10, GI254023X, before adding Atr-III. Whereas the inhibitor GI254023X alone did not affect GPVI shedding the ~55-kDa fragment of shedded GPVI ectodomain (sGPVI) appeared in the supernatant of Atr-III-pretreated platelets despite the presence of GI254023X. Therefore, these data suggest that Atr-III induces GPVI shedding independently of ADAM10.

**Figure 10:**
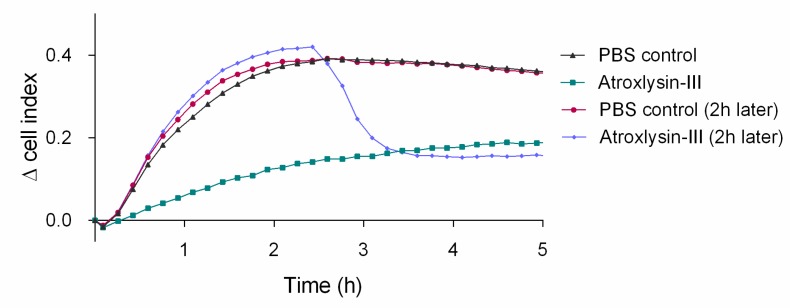
Platelet attachment to collagen-I-coated surface with concomitant and delayed (2 h) addition of Atr-III. The adhesion of platelets to collagen I was monitored by real-time impedance measurement in the xCELLigence system, on E-plates coated with 5 µg/mL of collagen I. Platelets treated with 32 µg/mL of Atr-III show a decreased adhesion, in both treatment regimens, at time 0 and 2 h after. The impedance values are corrected for the background values to obtain the real-time ∆ cell index values. This data is representative of three independent experiments performed in duplicates.

Moreover, Atr-III inhibited platelet interaction also to immobilized collagen-I, not only when added simultaneously with the platelets to the collagen-I but also when added 2 h after the seeded platelets had already adhered (Figure 10). In the first case, when Atr-III was added simultaneously with platelets the adhesion signal of platelets to collagen decreased throughout the entire observation period, likely due to degradation of the signaling collagen receptors. In the second case, when Atr-III was added to collagen-adherent platelets, the impedance value dropped drastically upon addition of Atr-III, indicating that Atr-III also acted on platelets after they had undergone the collagen-induced signaling and activation. This suggested that collagen receptors that mechanically anchored platelets to the collagen were degraded by Atr-III, resulting in detachment of already attached platelets and thrombi.

## 3. Discussion

Fibrino(geno)lytic metalloproteinases target critical biological systems such as blood coagulation, platelet function, and hemostasis. Anticoagulant SVMPs dissolve fibrin clots and avoid clot formation, thereby inducing severe bleeding and enhancing the toxic effects of hemorrhagic metalloproteinases. In this study, we purified and characterized a novel SVMP, Atr-III from the venom of the Peruvian pit viper snake *B. atrox*. The mature protein has a mass of around 56-kDa, comprises 420 amino acid residues and is structured into a M-domain of 200 residues, a D-domain of 101 residues, and a C-domain of 112 residues. The proprotein of pro-Atr-III has the consensus sequence PKMCGV that implies a Cys-switch mechanism for proteolytic activation of the SVMP upon maturation of SVMPs. Atr-III is a monomeric glycoprotein. Approx. 7% of its mol. mass is attributed to Asn-linked carboconjugates. Moreover, high Mr SVMPs which have larger carbohydrate moieties are usually more hemorrhagic [11]. In line with this, Atr-III causes hemorrhages. However, the molecular basis of how glycoconjugates affect the biochemical properties of P-III SVMPs is still unclear. They may stabilize the protein folding and, additionally, may influence binding and cleavage of substrate, as the N-linked glycoconjugate is located at Asn183 close to the Met-turn (Figure 3B), whose intrinsic flexibility has been proposed to be one of the critical decisive factors for SVMPs to cause hemorrhage [35]. Interestingly, the hemorrhagic P-I SVMP Atr-I has a non-glycosylable Asn residue at the homologous site of Asn183 of Atr-III.

The three-dimensional model of Atr-III was derived based on the VP2B structure of catrocolastatin (Figure 3A,B). More than 200 structures of metalloproteinases that have been deposited with the Protein Data Bank have revealed their common tertiary structure and active-site environment. However, each subfamily has distinct structural features. Similar to VP2B [34], Atr-III contains three Ca^2+^-binding sites, two of them (Ca^2+^-binding sites II and III)-within the D-domain located at “shoulder” (Ds) and “arm” (Da) segments, and another Ca^2+^-binding site opposite to the catalytic zinc atom and close to the C-terminus of the M-domain (Ca^2+^-binding site I) (Figure 3B). The C-domain is subdivided into “wrist” (Cw) and “hand” (Ch) segments (Figure 3B). Calcium ions likely stabilize the Atr-III structure, as proposed for other SVMPs/ADAMs and MMPs with collagenase activity. These proteinases are key participants in many, diverse immune and inflammation processes as well as in the remodeling of ECM [11,12]. Our sequence alignment of SVMPs (Figure 2) highlights that the number and spacing of cysteinyl residues are strictly conserved in the structure of SVMPs/ADAMs metalloproteinase family. Atr-III bears a disintegrin-like domain as the D and C domain are disulfide linked. The connecting cysteine within the disintegrin loop of Atr-III (SECD) is located in the generic XXCD motif of other SVMPs containing a disintegrin-like domain.

Atr-III is a member of the high Mr metalloproteinase family, consistent with the effects of Ca^2+^, Mg^2+^ and EDTA on its proteolytic activity [22,33,36,37,38]. Inhibition of proteolytic activity of Atr-III by adding Zn^2+^ may be explained by the hypothesis that Atr-III might have other Zn^2+^-binding sites with different binding affinity. The latter may be occupied by Zn^2+^ ions at high concentration, resulting in a conformational change and consequently loss of enzymatic function, as have been suggested for a P-III SVMP acurhagin from *A. acutus* venom [36]. To prove this hypothesis Atr-III was treated with different concentrations of Zn^2+^ before measured its proteolytic activity at pH 7.3. Regarding this point we observed a dose-dependent inhibition of DMC proteolysis by Atr-III with a complete blocked activity by adding 10 mM Zn^2+^ (Figure 5A,B). Elimination of Zn^2+^-binding ability of the respective histidines residues proves that the biological functions of Atr-III is mainly, but not necessarily, based on its proteolytic activity. The most detrimental among the pathophysiological effects of P-III SVMPs is the strong hemorrhagic activity, which disrupts either the process of hemostasis and or the interactions of cellular receptors and their ligands. Among the substrates of Atr-III are not only coagulation proteins, such as fibrin(ogen) but also ECM proteins of the vascular walls and adhesion receptors of platelets. It is particularly noteworthy that envenoming by *Bothrops* snakes are characterized by drastic hemostatic disturbances and inflammatory reactions with local tissue damage irradiating from the site of the bite [39]. Several studies demonstrate the ability of hemorrhagic metalloproteinases to degrade proteins of the basement membrane (BM) and other extracellular macromolecules using in vitro and in vivo strategies [38]. The broad substrate spectrum of Atr-III and other P-III SVMPs may be mediated not only by substrate recognition via the catalytic M domain but also by the non-enzymatic interaction of substrate with the non-proteinase domains (DC) in P-III SVMPs. The latter contain exosites that direct the interaction of these P-III SVMPs to specific ECM targets, especially in microvessels or in the plasma membrane of cells and platelets [37]. Furthermore, the presence of these domains may prevent the inhibition of these large metalloproteinases by endogenous inhibitors mainly by α2M in plasma or body fluids [34,38]. Owing to their multiple functions, the P-III SVMPs containing MDC domains found mostly in viper venoms species may serve as potential tools to better understand how these enzymes affect the mechanisms underlying thrombosis, hemostasis, and inflammatory reactions [23].

Our real-time adhesion results revealed that Atr-III impairs the interaction of platelets with collagen-I. The prerequisite for platelet adhesion to ECM proteins is platelet activation triggered by the collagen signaling receptors such as GPVI [24]. This leads to platelet spreading and a robust anchorage predominantly via α2β1 integrin. Therefore, several agents were designed to target platelet adhesion specifically, e.g., monoclonal antibodies directed against the GPVI receptor, and to interfere or temper the GPVI-collagen interaction [40]. Addition of Atr-III to platelets simultaneously while they are getting in contact with collagen-I affected and reduced signaling and platelet activation via GPVI. Distinctly, when adding Atr-III to the already adherent platelets, the detachment of adherent platelets and thrombi suggested that other collagen receptors of platelets may also be affected by Atr-III. Such a candidate target of Atr-III could be the anchoring receptor α2β1 integrin. We ruled out this hypothesis (data not shown) as the biochemical digestion assay revealed that only the integrin α2-subunit showed a slight, if not quantitatively negligible cleavage while the integrin β1-subunit seemed to be proteolytically robust against Atr-III. Moreover, the collagen-binding A-domain of α2β1 integrin was entirely resistant to Atr-III. The platelet collagen receptor GPVI contributes far more to the inhibitory effect of Atr-III on convulxin- and collagen-induced platelet aggregation than interaction with integrin α2β1. In this respect, the antiplatelet properties of Atr-III are similar to a homologous SVMP AAVI identified in *Formosan Agkistrodon acutus* venom that also function as GPVI antagonist [40]. It is of interest in this context that abnormally low levels of GPVI, caused by disease or deficiency, enhances its ADAM10-mediated shedding. This parameter together with elevated plasma sGPVI could be associated with an increase of risk of bleeding as observed in victims of viperid envenomation [41,42].

## 4. Materials and Methods

The venom was pooled from adults *B. atrox* specimens of both sexes, which had been captured in the rain forest of Alto Marañon (Peru) and kept in captivity in the serpentarium of the Natural History Museum, Universidad Nacional Mayor de San Marcos (UNMSM, Lima-Peru). Collagen type I (C9791), collagen type IV (C7521), laminin-111 from fibroblasts (L4544), fibronectin (F2006), marimastat (M2699), batimastat (SML0041), N-Ethylmaleimide (E3876), PMSF (78830), ADP (A2754), thrombin (112374), bovine (F8630), and human (F4129) fibrinogen essentially plasminogen free were obtained from Sigma Chemical (St. Louis, MO, USA). Type I collagen (5368), von Willebrand factor (vWF, 681300), ristocetin (001226) were from Helena Laboratories, Beaumont, TX, USA. *N*-glycosidase F (PNGase F—P0704S) and *O*-glycosidase (P0733S) were from New England Biolabs (Ipswich, MA, USA. MMP-III inhibitor (444264), Collagenase I inhibitor (234140), α2-Macroglobulin from human plasma (441251) were obtained from Calbiochem (San Diego, CA, USA). All other chemicals were of analytical reagent grade. All in vivo experiments were in accordance with the guidelines of the Brazilian College for Animal Experimentation and approved by the local Ethics Committee (Protocol number CEUA/Funed: 077/2015).

### 4.1. Purification of Atroxlysin III

Atr-III was purified by a three-step purification procedure. *B. atrox* venom (1.9 g), dissolved in 12 mL gel filtration buffer (50 mM ammonium acetate, pH 7.4, 0.3 M NaCl), was centrifuged at 6000× *g* and the supernatant (1.7 g protein) was separated on Sephacryl S-200 resin packed in a tandem of two (2.5 × 100 cm each) columns in the same buffer. The fractions containing proteolytic and hemorrhagic activities were pooled, dialyzed against a 1 mM CaCl_2_ solution in water containing and lyophilized. Thereafter, 272 mg of lyophilisate was dissolved in 2 mL 50 mM Hepes buffer, pH 8.0 containing 2 mM CaCl_2_ and purified on a DEAE-Sepharose CL-6B (1.6 × 17.5 cm) column with a linear salt gradient from 0–0.3 M NaCl at a flow rate of 13 mL/h. Proteolytically active and hemorrhagic fractions (35 mg) were pooled and separated by size exclusion on a Sephacryl S-200 (1.0 × 40 cm) column with 50 mM Hepes buffer, pH 8.0, containing 0.1 M NaCl and 2 mM CaCl_2_. Active metalloproteinase fractions containing atroxlysin-III (Atr-III) were pooled, dialyzed against distilled water and lyophilized.

### 4.2. MALDI-TOF Mass Spectrometry

MALDI-TOF-TOF spectra of Atr-III were recorded and analyzed using a Bruker Autoflex III Smartbean instrument (Billerica, MA, USA) in a linear positive mode controlled by the proprietary COMPASSTM 1.2 software package. The Nd-YAG-laser power (355 nm) was manually adjusted for optimal signal appearance. A freeze-dried 0.5 μL solution of salt- and detergent free protein in 30% ACN in 0.1% TFA was spotted on the ground steel target plate, mixed with 0.5 μL 10 mg/mL sinapinic acid in 50% ACN, 0.1% TFA and left to dry at room temperature (RT), next to standard protein mixtures for calibration.

### 4.3. Glycosylation Studies

The carbohydrate content of Atr-III was confirmed with PNGase F and O-glycosidase according to the manufacturer’s instructions. To determine N-linked sugars, 10 μg of Atr-III were added to denaturing buffer, boiled for 10 min, and subsequently treated with glyco buffer, NP 40 (1%) and 2 units of recombinant PNGase F or O-glycosidase for 1 h at 37 °C. The reaction was terminated by boiling for 5 min. After addition of PAGE loading buffer, the reaction mixture, along with untreated control and deglycosylated enzyme, were analyzed by SDS-PAGE. To maintain the activity of Atr-III, the enzyme was also deglycosylated with PNGase F for 1 h at 37 °C in the absence of reducing and denaturing agents [43].

### 4.4. Enzymatic Features of Atr-III

Proteolytic activity of Atr-III was assessed with the substrate DMC [44]. To test the effects of cations, Atr-III (1 μg) was pretreated with CaCl_2_, MgCl_2_ and ZnCl_2_ (1 mM each) in 25 mM Hepes buffer, pH 7.4 for 30 min at 37 °C before enzymatic activity was quantified by DMC digestion. DMC proteolysis of Atr-III (1 μg) in 250 μL of 25 mM Hepes buffer, pH 7.4 for 5 min at 37°C was also measured in the presence of several proteinase inhibitors: EDTA, PMSF (1 mM each), I-Coll-I, I-MMP-III (ratio of 1:50 each), synthetic MMP inhibitors: BAT and MAR (0.5 µM each), as well as the main plasma inhibitor α2-M (7.5–60 μg; molar enzyme:inhibitor ratios: 0.2–2.4).

### 4.5. Modification of Histidine Residues in Atr-III

The essential histidine residues of Atr-III were chemically modified to prevent Atr-III from complexing Zn^2+^. Thereby, the enzyme is persistently inactivated even after restoration with Zn^2+^. Modification of histidine with DEPC was carried out according to [45]. To this end, 20 μL Atr-III (1 mg/mL) in 80 μL PBS (0.01 M NaH_2_PO_4_, 0.15 M NaCl, pH 6.5) containing 0.4 μL of 0.5 mM EDTA was mixed with 4 μL of 130 mM DEPC/waterfree acetonitrile (ACN) (5 mM final concentration DEPC and 3.8% ACN), incubated at 15 °C for 1 h in thermomixer (300 rpm), followed by four dialysis rounds in micro dialysis tubes against PBS, pH 7.4 for 30 min each. Proteolytic activities of treated and untreated Atr-III (4 µg each) were tested with 40 µg Fg (1.3 mg/mL) in PBS, pH 7.4, containing 2 mM CaCl_2_ (2 µL) and 5 mM Zn-acetate (5 µL) to final volume of 100 µL. Samples were drawn after certain time intervals and the digestion was stopped by immediately adding 1 µL of EDTA (12 mM) and cooling on ice. TCA precipitated and acetone-washed samples were separated by SDS-PAGE (5–10% gradient) under reducing conditions and the gel stained with Coomassie.

### 4.6. Degradation of Plasma and ECM Proteins by Atr-III

Proteolytic activity of Atr-III was also investigated on human Fg, fibrin, and fibronectin (FN). To this end, Fg was dissolved at a final concentration of 2.5 mg/mL in 25 mM Tris-HCl, pH 7.4 containing 154 mM NaCl. Atr-III (2 μg) was added to 100 μL of Fg solution at a molar enzyme:substrate-ratio of 1:150 and the reaction was incubated at 37 °C. At time intervals (5, 15, 30, and 60 min), reactions were stopped by added 40 μL of denaturing solution (10 M urea, 4% β-mercaptoethanol, 4% SDS), and analyzed by SDS-PAGE (12% gel). The fibrinolytic activity was tested using the fibrin plate method [44]. The fibrin gels were produced by adding 2 NIH U/mL of human thrombin (Sigma) to bovine Fg solution (2.5 mg/mL) in Tris-HCl buffered saline, pH 7.4, containing 154 mM NaCl and by incubating 2 h at RT. Different amounts of Atr-III in a 10 μL volume were then added and incubated for 15 h at 37 °C. The areas of lysis were measured by flooding the plate with 10% TCA solution. Protein-chemically, degradation of fibrin and other Atr-III substrates was analyzed by SDS-PAGE (12% gel) at different time points of digestion. Other substrates of Atr-III were plasma FN, and the ECM proteins laminin-111 (LM), type I and IV collagens at an enzyme:substrate-ratio of 1:100 (FN) and 1:50, respectively. Digestion was carried out at 37 °C, except for type IV collagen at 25 °C.

### 4.7. Effect of Atr-III on α2β1 Integrin, on its Recombinant α2A-Domain (rα2A-Domain) and on Recombinant von Willebrand Factor A1-domain (rWF-A1 Domain)

The recombinant ectodomain of integrin α2β1 [46] and its rα2A-domain were incubated with Atr-III (10 μg) in 50 mM PBS, pH 7.4 at 37 °C for 5, 15, 30, and 60 min in the absence or presence of 10 mM EDTA, the former ones stopped with 10 mM EDTA and analyzed with SDS-PAGE (7–15% gradient gel). Controls of Atr-III and the undigested integrin or rα2A-domain alone were included. The rvWF-A1-domain (5 µg) was digested with Atr-III (0.5 µg) or incubated alone according to [14].

### 4.8. Synthesis and Sequencing of cDNA

The cDNA was obtained from fresh venom of an adult male *B. atrox* specimen as described [47]. The gene of Atr-III was amplified with the kit Master Mix Platinum^®^Taq DNA Polymerase (Invitrogen, Carlsbad, CA, USA) according to the manufacturer’s instructions. The external primers MpIIIF: 5′gaactcagattggcttgaagga3′ and MpIIIR: 5′ggaagtagctacatcttggaaagcc3′(kindly provided by Prof. J. Mendoza, Facultad de Ciencias Biologicas, UNMSM) were designed on the basis of the highly conserved cDNA sequences encoding P-III SVMPs from *Bothrops* species, bothropasin (*B. jararaca*, AF056025.2), *B.* neuwiedi MP-III3 (HM443634.1), jararhagin (*B. jararaca*, X68251.1), berythractivase (*B. erythromelas*, AF450503.1), *B. jararacussu* (RGD-P-III MP DQ408681.1), and *B. insularis* MP (AF490534.1). Sequencing of the amplification products was performed on an ABI 3730 XL automated sequencer (Macrogen, Inc, South Korea) using the internal primers MpIIIFi: 5′ attgttgaggattatagcccaat 3′ and MpIIIRi5′ tgcttatcgtgggagccataatt 3′. The obtained protein sequences were compared with sequences deposited in GenBank and SwissProt.

### 4.9. Multiple Alignment and Phylogenetic Tree Construction

Sequence alignments were performed using CLUSTALW multi-sequence alignment program from Bioedit [48]. To examine the evolutionary relationship among SVMPs, a phylogenetic tree was built using the MEGA program v. 6.0 [49] according to the neighbor-joining method. The phylogenetic distance was calculated by the method of Kimura using two parameters and performing 1000 replicates (bootstraps).

### 4.10. Modeling of Atr-III Three-dimensional Structure

A three-dimensional model of Atr-III was predicted using comparative homology modeling [50] based on appropriate homologue templates found with a BLAST search: *B. jararaca* (PDB code 3DSL|chain B), *C. atrox* (PDB code 2DWO|chainA) and *A. acutus* (PDB code 3HDB|chainA) showing 97%, 86%, and 84% identity, respectively, and 99% query coverage with each one. Starting from these selected templates, 10 models were calculated by the program, Modeller 9.2. RMS deviations VMD v 1.9.2. Stereochemistry of the predicted model was checked by PROCHECK [51]. Metal-binding sites were analyzed by IONCOM server (http://zhanglab.ccmb.med.umich.edu/IonCom/). PyMOL, v.2002 (http://www.pymol.org) was used to illustrate the structure models.

### 4.11. Platelet Aggregation Assay

Human blood from healthy volunteers was collected in acid-citrate-dextrose (ACD: 78 mM citric acid; 117 mM sodium citrate; 282 mM dextrose) [6:1, (*v*/*v*)] centrifuged at 200× *g* for 15 min to obtain platelet-rich-plasma (PRP). Washed platelets were isolated as described [14]. Washed platelets were re-suspended in Tyrode’s solution, pH 7.4, containing 2 mM CaCl_2_ and 1 mM MgCl_2_ in absence of prostaglandin E1 (PGE1). Platelet density was adjusted to 2.5 × 10^5^ platelets/µL. Platelet aggregation was carried out in an eight channel platelet aggregometer (AggRam Helena Laboratories, Beaumont, TX, USA) with stirring (600 rpm) at 37 °C. To examine the effect of Atr-III on platelets, washed platelets (225 µL) were pre-incubated with several concentrations of Atr-III (2 to 32 µg/mL) in Tyrode’s solution pH 7.4, for 3 min. Platelets were stimulated by addition of different agonists: 6 µg/mL of convulxin (CVX) isolated from *Crotalus durissus terrificus* snake venom, 10 µg/mL of collagen-I, 7 µg/mL of vWF plus 0.5 mg/mL of ristocetin. Light transmittance was recorded and the inhibition of platelet aggregation was measured at the maximum aggregation response.

### 4.12. Western Blot Assays

The possible metalloproteinase degradation of GPIbα and shedding of GPVI from the surface of washed platelets by Atr-III, was analyzed by immunoblotting assay using specific antibodies. To test GPIbα degradation, washed platelets (5 × 10^8^ cells/mL) were incubated with Atr-III (10 µg) for 5, 15, 30, or 60 min at 37 °C. The reaction was stopped by addition of SDS sample loading buffer. To assess GPVI shedding, 225 µL of washed platelets (5 × 10^8^ cells/mL) were incubated with Atr-III (10 µg) at the above conditions. To gain information on the involvement of ADAM10 in GPVI shedding, platelets were incubated with GI254023X 100 nM (3995, Tocris), an specific inhibitor of ADAM10 with or without Atr-III (10 µg), and NEM (*N*-ethylmaleimide) (10 mM) as positive control, for 60 min at 37 °C. To isolate platelets from incubation medium supernatants, platelets were pelleted by centrifugation at 15,000× *g* for 2 min. The supernatant fraction was isolated from pellet and both were mixed with SDS sample loading buffer. All aliquots were separated by SDS-PAGE, transferred to a nitrocellulose membrane and probed with anti-human GPVI polyclonal antibody (AF3627, R&D Systems), anti-human CD42b/GPIbα polyclonal antibody (AF4067, R&D Systems), or anti-β-actin (IM-0075, Imuny) and a secondary alkaline-phosphatase conjugated antibody (Sigma-Aldrich).

### 4.13. Platelet Adhesion Assays Using xCELLigence Technology

The effect of Atr-III on platelets adhesion was quantified by the label-free, impedance-based real-time technology (xCelligence System). To this end, an E-plate was coated with 5 μg/mL of collagen I at 4 °C overnight. Non-specific binding was blocked with heat-inactivated BSA at a concentration of 1%. The platelets were seeded at a density of 50,000/μL, and treated with Atr-III (32 μg/mL) either simultaneously (time 0) or 2 h later. PBS, instead of Atr-III, was added to the control well. The impedance change depends on the number of attached platelets and their morphometric changes.

## Figures and Tables

**Table 1:** Some biological activities of Atroxlysin-III from *B. atrox* venom.

	Proteolytic (U/mg) ^1^	Hemorrhagic (U/mg) ^2^	Fibrinolytic (U/µg) ^3^ Bovine Fibrin
**Crude venom**	1.45 ± 0.39	68.9 ± 2.26	7.33 ± 0.65
**Atr-III**	27.8 ± 2.47	4000 ± 1.54	8.43 ± 0.06

^1^ One unit is defined as ΔA340/min. Specific proteolytic activity is expressed as U/mg protein using dimethylcasein (DMC) as substrate. ^2^ One unit of hemorrhagic activity is one minimum hemorrhagic dose (MHD). Specific activity is expressed as U/mg protein (13). ^3^ One unit is defined as lyzed zone (mm) per µg protein.

## Supplementary Materials

The following supplementary information are available online, Figure S1: Purification of Atroxlysin-III from *B. atrox* venom (1st and 2nd step), Figure S2: The nucleotide and deduced amino acid sequence of Atr-III precursor from *B. atrox* venom, Figure S3: Effect of Atr-III on platelet aggregation stimulated with convulxin (CVX), collagen or von Willebrand factor plus ristocetin.

## References

[1] Casewell N.R., Wüster W., Vonk F.J., Harrison R.A., Fry B.G. (2013). Complex cocktails: The evolutionary novelty of venoms. Trends Ecol. Evol..

[2] Fry B.G., Vidal N., Norman J.A., Vonk F.J., Scheib H., Ramjan S.F., Kuruppu S., Fung K., Hedges S.B., Richardson M.K. (2006). Early evolution of the venom system in lizards and snakes. Nature.

[3] Calvete J.J. (2017). Venomics: Integrative venom proteomics and beyond. Biochem. J..

[4] Lu Q., Clemetson J.M., Clemetson K.J. (2005). Snake venoms and hemostasis. J. Thromb. Haemost..

[5] Escoubas P., King G.F. (2009). Venomics as a drug discovery platform. Expert Rev. Proteom..

[6] Marsh N. (2005). Practical applications of snake venom toxins in hemostasis. Toxicon.

[7] Markland F.S., Swenson S.D., Gopalakrishnakone P., Calvete J.J. (2016). Application of snake toxins in biomedicine. Venom Genomics and Proteomics, Toxinology.

[8] Calvete J.J., Borges A., Segura A., Flores-Dias M., Alape-Giron A., Gutierrez J.M., Diez N., De Souza L., Kiriokos D., Sanchez E. (2009). Snake venomics and antivenomics of *Bothrops colombiensis*, a medically important pitviper of the *Bothrops atrox-asper* complex endemic to Venezuela: Contributing to its taxonomy and snake bite management. J. Proteom..

[9] Kohlhoff M., Borges M.H., Yarleque A., Cabezas C., Richardson M., Sanchez E.F. (2012). Exploring the proteomes of the venoms of the Peruvian pit vipers *Bothrops atrox*, *B. barnetti* and *B. pictus*. J. Proteom..

[10] Takeda S., Takeya H., Iwanaga S. (2012). Snake venom metalloproteinases: Structure, function and relevance to the mammalian ADAM/ADAMTS family proteins. Biochim. Biophys. Acta.

[11] Fox J.W., Serrano S.M. (2008). Insights into speculations about sanke venom metalloproteinase (SVMP) synthesis, folding and disulfide bond formation and their contribution to venom complexity. FEBS J..

[12] Takeda S. (2016). ADAM and ADAMTS family proteins and snake venom metalloproteinases: A structural overview. Toxins.

[13] Sanchez E.F., Schneider F.S., Yarleque A., Borges M., Richardson M., Figueiredo S.G., Evangelista K., Eble J.A. (2010). The novel metalloproteinase atroxlysin-I from Peruvian *Bothrops atrox* (Jergón) snake venom acts both on ECM and platelets. Arch. Biochem. Biophys..

[14] Sanchez E.F., Richardson M., Gremski L.H., Veiga S.S., Yarleque A., Niland S., Lima A.M., Estevao-Costa M.I., Eble J.A. (2016). A novel fibrinolytic metalloproteinase, barnettlysin-I from *Bothrops barnetti* (barnett’s pitviper) snake venom with anti-platelet properties. Biochim. Biophys. Acta..

[15] You W.-K., Jang Y.-J., Chung K.-H. (2006). Functional roles of two distinct domains of halysase, a snake venom metalloprotease, to inhibit human platelet aggregation. Biochem. Biophys. Res. Comm..

[16] Gibeler N., Zigrino P. (2016). A disintegrin and metalloprotease (ADAM): Historical overview of their functions. Toxins.

[17] Lu X., Lu D., Scully M.F., Kakkar V.V. (2005). Snake venom metalloproteinase containing a disintegrin-like domain, its structure-activity relationships at interacting with integrins. Curr. Med. Chem. Cardiovasc. Hematol. Agents.

[18] Tanjoni I., Evangelista K., Della-Casa M.S., Butera D., Magalhães G.S., Baldo C., Clisa P.B., Fernandes I., Eble J., Moura da Silva A.M. (2010). Different regions of the class P-III snake venom metalloproteinase jararhagin are involved in binding to α2β1 integrin and collagen. Toxicon.

[19] Gardiner E.E. (2017). Proteolytic processing of platelet receptors. Res. Pract. Thromb. Haemost..

[20] Gardiner E.E., Andrews R.K. (2014). Platelet receptor expression and shedding: Glycoprotein Ib-IX-V and glycoprotein VI. Transfus. Med. Rev..

[21] Ruggeri C.M. (2002). Platelets in atherothrombosis. Nat. Med..

[22] Wang W.-J., Shih C.-H., Huang T.-F. (2005). Primary structure and antiplatelet mechanism of snake venom metalloproteinase, acurhagin, from *Agkistrodon acutus* venom. Biochimie.

[23] Kamiguti A.S. (2005). Platelets as targets of snake venom metalloproteinases. Toxicon.

[24] Wijeyewickrema L.C., Gardiner V., Moroi M., Berndt M.C., Andrews R.K. (2007). Snake venom metalloproteinases, crotarhagin and alborhagin, induce ectodomain shedding of platelet collagen receptor, glycoprotein VI. Thromb. Haemost..

[25] Nuñez V., Cid P., Sanz L., La Torre P.D., Angulo Y., Lomonte B., Gutierrez J.M., Calvete J.J. (2009). Snake venomics and antivenomics of *Bothrops atrox* venoms from Colombia and the Amazon regions of Brazil, Perú and Ecuador suggest the occurrence of geographic variation of venom phenotype by a trend towards paedomorphism. J. Proteom..

[26] Calvete J.J., Sanz L., Perez A., Borges A., Vargas A.M., Lomonte B., Angulo Y., Gutierrez J.M., Chalkidis H.M., Mourão R.H.V. (2011). Snake population venomics and antivenomics of *Bothrops atrox*: Paedomorphism along its transamazonian dispersal and implications of geographic venom variability on snakebite management. J. Proteom..

[27] Warrell D.A., Campbell J.A., Lamar W.W. (2004). Snakebites in Central and South America: Epidemiology, clinical features and clinical management. The Venomous Reptiles of the Western Hemisphere.

[28] Zavaleta A. (2004). Mordedura de serpiente (Ofidismo): Un problema de salud en el Perú. Rev. Med. Hered..

[29] Paine M.J.I., Desmond H.P., Theakston R.D.G., Crampton J.M. (1992). Purification, cloning and molecular characterization of a high molecular weight hemorrhagic metalloproteinase, jararhagin from *Bothrops jararaca* venom. J. Biol. Chem..

[30] Assakura M.T., Silva C.A., Mendele R., Camargo A.C.M., Serrano S.M.T. (2003). Molecular cloning and expression, of structural domains of bothropasin, a P-III metalloproteinase from the venom of *Bothrops jararaca*. Toxicon.

[31] Freitas-de-Sousa L.A., Amazonas D.R., Souza L.F., San’tAnna S.S., Nishiyama M.Y., Serrano S.M.T., Junqueira-de Azevedo I.L., Chalkidis H.M., Moura-da-Silva A.M. (2015). Comparisons of venoms from wild and long-term captive *Bothrops atrox* snakes and characterization of batroxrhagin, the predominant class P-III metalloproteinase from the venom of this species. Biochimie.

[32] Junqueira-de-Azevedo I.L., Ho P.L. (2002). A survey of gene expression and diversity in the venom glands of the pitviper snake *Bothrops insularis* throught the generation of expressed sequence tags (ESTs). Gene.

[33] Sanchez E.F., Gabriel L.M., Gontijo S., Gremski L.H., Veiga S.S., Evangelista K., Eble J.A., Richardson M. (2007). Structural and functional characterization of a P-III metalloproteinase, leucurolysin-B, from *Bothrops leucurus* venom. Arch. Biochem. Biophys..

[34] Igarashi T., Araki S., Mori H., Takeda S. (2007). Crystal structures of catrocolastatin/VAP2B reveal a dynamic, modular architecture of ADAM/adamalysin/reprolysin family proteins. FEBS Lett..

[35] Wallnoefer H.C., Lingott T., Gutierrez J.M., Merfort I., Liedl K.R. (2010). Backbone flexibility controls the activity and specificity of a protein-protein interface: Specificity in snake venom metalloproteases. J. Am. Chem. Soc..

[36] Wang W.-J., Huang T.-F. (2002). Purification and characterization of a novel metalloproteinase, acurhagin, from *Agkistrodon acutus* venom. Thromb. Haemost..

[37] Serrano S.M.T., Wang D., Shannon J.D., Pinto A.F.M., Polanowska-Grabowska R.K. (2007). Interaction of the cysteine-rich domain of snake venom metalloproteinases with the A1 domain of von Willebrand factor promotes site-specific proteolysis of von Willebrand factor and inhibition of von Willebrand factor-mediated platelet aggregation. FEBS J..

[38] Herrera C., Voisin M.B., Escalante T., Rucavado A., Nourshargh S., Gutierrez J.M. (2016). Effects of PI and PIII snake venom haemorrhagic metalloproteinases on the microvasculature: A confocal microscopy study on the mouse cremaster muscle. PLoS ONE.

[39] Bernardoni J.L., Sousa L.F., Wermelinger L.S., Lopes A.S., Prezoto B.C., Serrano S.M.T., Zingali R.B., Moura-da-Silva A.M. (2014). Functional variability of snake venom metalloproteinases: Adaptive advantages in targeting different prey and implications for human envenomation. PLoS ONE.

[40] Wang W.-J. (2007). Purification and functional characterization of AAVI, a novel P-III metalloproteinase, from Formosan *Agkistrodon acutus* venom. Biochimie.

[41] De Meyer S.F., Vanhoorelbeke K., Broos K., Salles I.I., Deckmyn H. (2008). Antiplatelet drugs. Br. J. Haematol..

[42] Montague S.J., Andrews R.K., Gardiner E.E. (2018). Mechanisms of receptor shedding in platelets. Blood.

[43] Oliveira A.K., Leme A.F.P., Asega A.F., Camargo A.C.M., Fox J.W., Serrano M.T.S. (2010). New insights into the structural elements involved in the skin haemorrhage induced by snake venom metalloproteinases. Thromb. Haemost..

[44] Sanchez E.F., Santos C.I., Magalhaes A., Diniz C.R., Figueiredo S., Gilroy J., Richardson M. (2000). Isolation of a proteinase with plasminogen-activating activity from *Lachesis muta muta* (bushmaster) snake venom. Arch. Biochem. Biophys..

[45] Miles E.W. (1977). Modification of histidyl residues in proteins by diethylpyrocarbonate. Methods Enzymol..

[46] Eble J.A., Beermann B., Hinz H.-J. (2001). α2β1 integrin is not recognized by rhodocythin but is the specific high affinity target of rodochetin, an RGD-independent disintegrin and potent inhibitor of cell adhesion to collagen. J. Biol. Chem..

[47] Vivas-Ruiz D.E., Sandoval G.A., Mendoza J., Inga R.R., Gontijo S., Richardon M., Eble J.A., Yarleque A., Sanchez E.F. (2013). Coagulant thrombin-like enzyme (barnettobin) from *Bothrops barnetti* venom: Molecular sequence analysis of its cDNA and biochemical properties. Biochimie.

[48] Hall T.A. (1999). BioEdit: A user-friendly biological sequence alignment editor and analysis program for Windows 95/98/NT. Nucl. Acids Symp. Ser..

[49] Tamura K., Stecher G., Peterson D., Filipski A., Kumar S. (2013). MEGA6: Molecular Evolutionay Genetics Analysis Version 6.0. Mol. Biol. Evol..

[50] Sali A., Blundell T.L. (1997). Comparative protein modelling by satisfaction of spatial restraints. J. Mol. Biol..

[51] Laskowski R.A., Macarthur M.W., Moss D.S., Thorton J.M. (1993). PROCHECK: A program to check the stereochemical quality of protein structures. J. Appl. Cryst..

