# Supplementary material for: Atroxlysin-III, A Metalloproteinase from the Venom of the Peruvian Pit Viper Snake Bothrops atrox (Jergón) Induces Glycoprotein VI Shedding and Impairs Platelet Function
Source: Molecules. 2019 Sep 26;24(19):3489. doi: 10.3390/molecules24193489 (PMC6803841; doi:10.3390/molecules24193489)
Supplement: Supplementary file 1 [file molecules-24-03489-s001.pdf]

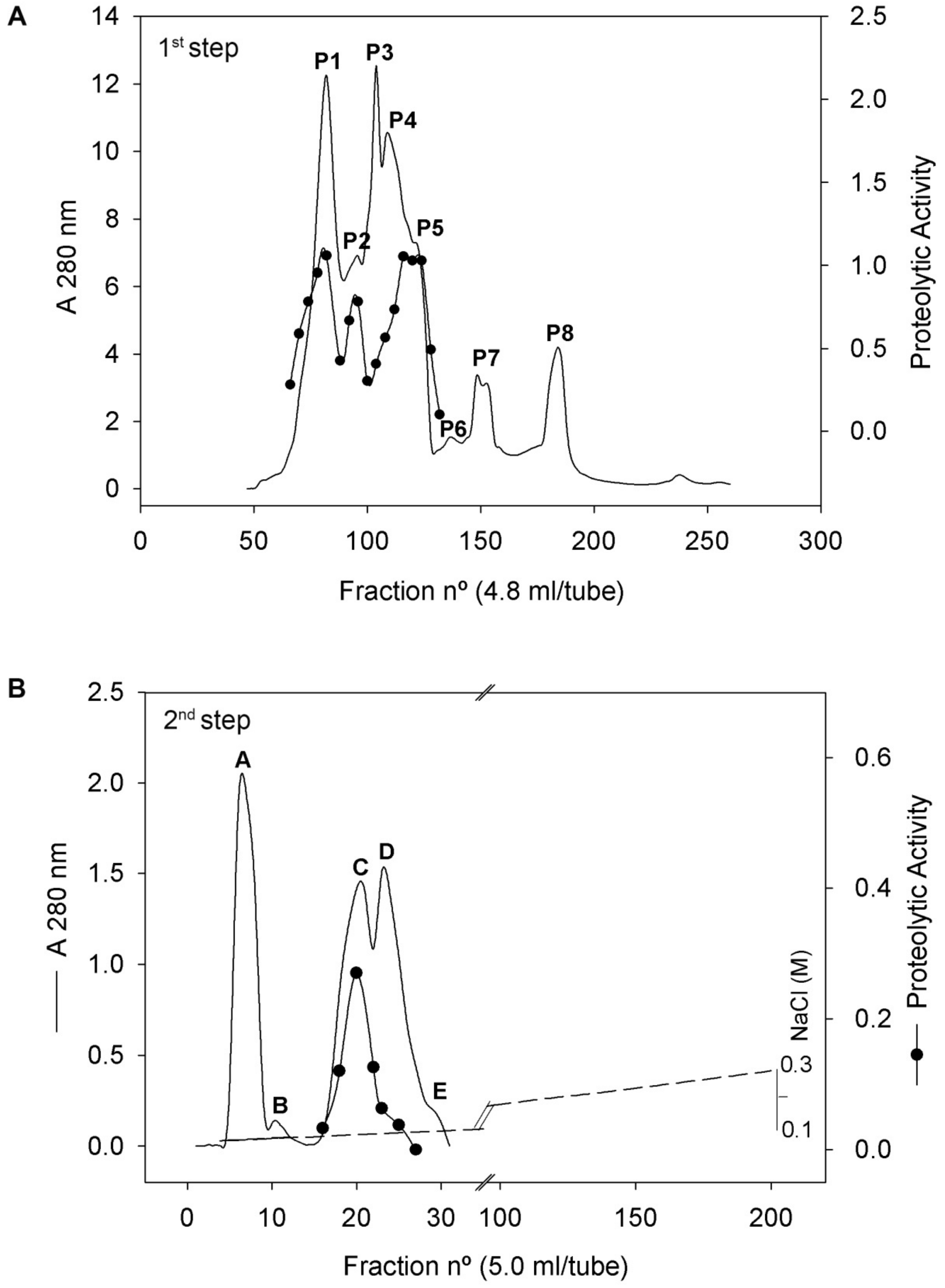

Figure 1. Purification of Atroxlysin-III from *B. atrox* venom. (**A**) Crude venom of *B. atrox* was applied to a Sephacryl 200 column. The column was equilibrated and eluted with 50 mM ammonium acetate buffer, pH 7.5, containing 0.3 M NaCl at a flow rate of 7.2 mL/h. The peak P1 (with proteolytic and hemorrhagic activity) was applied to a DEAE-ion exchange column. (**B**) The DEAE Sepharose CL-6B column was equilibrated with 50 mM Tris-HCl buffer, pH 8.0, and eluted with a linear salt gradient from 0–0.3 M NaCl at a flow rate of 13 mL/h. Peak C contained the hemorrhagic and proteolytic activity.

aagagattgcctggcttccagccaaatccagcctccaaaatgattcatgttctcttggtc

K R L P G F Q P N P A S K **M I H V L L V**

actatatgcttagcagcttttccttatcaaggagctctataatcctggaatctgggaac

T I C L A A F P Y Q G S S I I L E S G N

gtgaatgattatgaagtagtgtatccacgaaaagtcactgcattgcccaaaggagcaatt

V N D Y E V V Y P R K V T A L P K G A I

caaccaaagtatgaagacaccatgcaatatgaatttaaagtgaatggagagccagtggtc

Q P K Y E D T M Q Y E F K V N G E P V V

cttcacctggaaaaaaataaaggactttttcaaaagattacagcgagattcattattcc

L H L E K N K G L F S K D Y S E I H Y S

cctgatggcagaaaaattacaacataccccccggttgaggatcactgctattatcatgga

P D G R K I T T Y P P V E D H C Y Y H G

cccatcaagaatgatgctgactcaactgcaagcatcaatgcatgctacggtttgaaagga

P I K N D A D S T A S I N A C Y G L K G

tatttcaagcttcaaagggacacgtactttattgaacccttgaagcttcccgacagtgaa

Y F K L Q R D T Y F I E P L K L P D S E

gcccatgcagtcttcaaatatgaaaatgtagaaaaggaggatgaggcccccaaaatgtgt

A H A V F K Y E N V E K E D E A ***P K M C***

ggggtaacccagaattggaaatcatatgaacccatcaaaaaggcctctcatttagctttt

G V T Q N W K S Y E P I K K A S H L A F

actgctgaacaacaaaaatataaccccttcagatacgttgagtttctcttagttgtggac

T A E Q Q K Y N P F R Y V E F L L V V D

caaggaatgctcacaaaaaacaatggcgatttaaataaaataaaatcaagaatgtctgaa

Q G M L T K N N G D L N K I K S R M S E

cttgccaaaattgtgaatgagattttaagatacctgtatttgcatgctgcactggttggc

L A K I V N E I L R Y L Y L H A A L V G

ctacaaatttggaccaatggagataagattaccgtgaagccggacgtggattatactttg

L Q I W T N G D K I T V K P D V D Y T L

aattcatttgcagaatggagaaaaacagatttgctgactcgcaaaaaacatgataatgct

N S F A E W R K T D L L T R K K H D N A

cagttactcacagcaattgacttcaatggaccaactataggatatgcttacataggcagc

Q L L T A I D F N G P T I G Y A Y I G S

atgtgccacccgaagcgttctgtagcaattgttcaggattatagcccaataaatcttgtg

M C H P K R S V A I V Q D Y S P I N L V

gttgctgttataatggcccatgagatgggtcataatctgggcattcatcatgacacagat

V A V I M A **H E M G H N L G I H H D** T D

ttctgttcttgtggtgattacccatgcattatgggtcccacgataagcaatgaaccttcg

F C S C G D Y P **C I M** G P T I S N E P S

aaatttttcagcaattgtagttatatccaatgttgggactttattatgaaggacaaccca

K F F S N C S Y I Q C W D F I M K D N P

caatgcattatcaatgaacccttgggaacagatattgtttcacctccagtttgtggaaat

Q C I I N E P L G T D I V S P P V C G N

gaacttttggaggtgggagaagaatgtgactgtggcactcctgaaaattgtcaaaatgag

E L L E V G E E C D C G T P E N C Q N E

tgctgcgatgctgcaacgtgtaaactgaaatcagggtcacagtgtggacatggagactgt

C C D A A T C K L K S G S Q C G H G D C

tgtgagcaatgcaaatttagcaaatcaggaacagaatgccgggcatcaatgagtgaatgt

C E Q C K F S K S G T E C R A S M S E C

gacccggctgaacactgcactggccaatcttctgagtgtcctgcagatgtcttccataag

D P A E H C T G Q S S E C P A D V F H K

aatggacaaccatgcctagataactacggttactgctacaatgggaattgccccatcatg

N G Q P C L D N Y G Y C Y N G N C P I M

tatcaccaatgttatgctctctttggtgcagatgtttatgaggctgaagattcatgcttc

Y H Q C Y A L F G A D V Y E A E D S C F

aaagataaccagaaaggcaattattatggctactgcagaaaggaaaatggtaaaaagatt

K D N Q K G N Y Y G Y C R K E N G K K I

```
ccatgtgcaccagaagatgtaaaatgtggcaggttatactgcaaagataattcacctgga
 P  C  A  P  E  D  V  K  C  G  R  L  Y  C  K  D  N  S  P  G
caaaataatccttgcaagatgttctattccaacgatgatgaacataagggaatggttctt
 Q  N  N  P  C  K  M  F  Y  S  N  D  D  E  H  K  G  M  V  L
cctggaacaaaatgtgcagatggaaaggtgtgcagcaacgggcattgtgttgatgtggct
 P  G  T  K  C  A  D  G  K  V  C  S  N  G  H  C  V  D  V  A
acagcctactagtcaacctctggctttgattttggagatcctccttccagaaggtttggc
 T  A  Y  *
ttctctcaagtccaaagagatccatctgcctgcatcttactagtaaatcactcttagctt
ccagatggcatctaaattctgcaatatttcttcaccatatttaatctgtttacctttttgc
tgtaatcaaacctttttccccaccacaaagctccatgggcatgtacaacaccaaaggctta
tttgctgtcaagaaaaaaaaatggccattttataccgtttgccaattgcagagcacattt
aatacaagaagttctgccttttgagctggtgtattcgaagtcaatgcttcctctcccaaa
attttgtgctggctttccaaaatgtagctacttccatcaataa
```

Figure 2. The nucleotide and deduced amino acid sequence of Atr-III precursor from *B. atrox* venom. The predicted amino acid sequence is shown above the nucleotide sequence: sequence starts with the amino terminal amino acid of mature protein (glutamic acid). A signal peptide (MIHVLLVTICLAAFPYQG), a cysteine-switch motif (PKMCGV) are bold-underlined. The Zinc binding motif (HEGNHLGIHHD) and the methionine 168 of the Met-turn region are invariant and are highlighted in blue. Putative N-linked glycosylation site is bold and boxed in yellow. Disintegrin-like (ECD) sequences are highlighted in green. Termination codon and polyadenylation addition signal (aataa) are denoted. Accession number of Atroxlysin-III precursor in the GenBank database is KX821773.

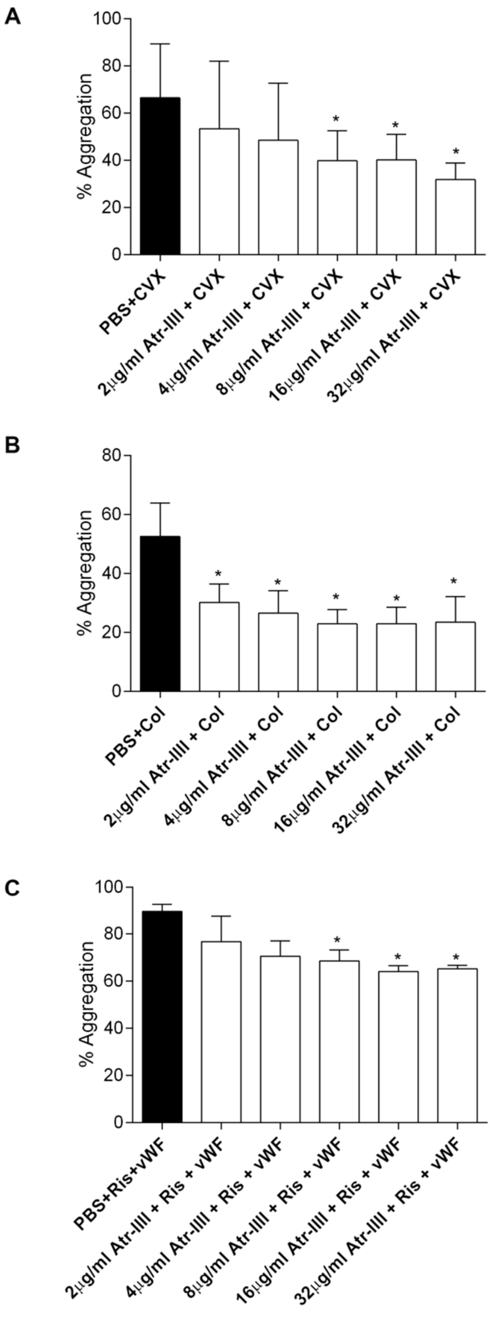

Figure S3: Washed human platelets (225 μL, $2.5x10^5$/μL) were pre-incubated with different concentrations of Atr-III: 2 to 32 μg/mL) for 3 minutes and stirred (600 rpm) at 37 °C. After incubation, platelets were stimulated with different agonists: (A) 6 μg/mL of convulxin (CVX), (B) 10 μg/mL of collagen-I, (C) 5 μg/mL of human vWF plus 0.5mg/mL of ristocetin. Black bars represent the positive control (platelets pre-incubated with 10 μL PBS). *P < 0.05 compared with PBS plus agonist (1-way ANOVA followed by Tukey's test n = 4).
